# Conjunctival flora of clinically normal and diseased turtles and tortoises

**DOI:** 10.1186/s12917-015-0405-x

**Published:** 2015-04-10

**Authors:** Francesco Di Ianni, Pier Luigi Dodi, Clotilde Silvia Cabassi, Igor Pelizzone, Andrea Sala, Sandro Cavirani, Enrico Parmigiani, Fausto Quintavalla, Simone Taddei

**Affiliations:** Dipartimento di Scienze Medico-Veterinarie, Università di Parma, via del Taglio 10, 43126 Parma, Italy

**Keywords:** Turtles, Conjunctiva, Microbial flora, Zoonosis

## Abstract

**Background:**

In captive breed turtles and tortoises conjunctival disease is common. Our aim was to investigate the bacterial and fungal flora present in the eyes of healthy and pathological chelonians and to compare findings in turtles with those in tortoises.

**Results:**

Samples were taken from the conjunctival sacs of 34, diseased and healthy, chelonians (18 tortoises and 16 turtles) and submitted to bacterial and fungal investigation. All samples showed bacterial growth. Thirteen animals (38%), harboured a single bacterial species as sole isolate and twenty-one animals (62%) harboured more than one species. Detection of multiple bacterial infection was clearly greater in tortoises compared to turtles. Most frequently isolated bacterial species were *Bacillus* spp. (13 isolates), *Staphylococcus xylosus* (10 isolates), *Sphingomonas paucimobilis* (6 isolates), *Staphylococcus sciuri* and *Aeromonas hydrophila/caviae* (each 5 isolates), *Ochrobactrum anthropi* (3 isolates), *Citrobacter freundii*, *Enterobacter cloacae* and *Pseudomonas luteola* (each 2 isolates). Only one isolate of *Kocuria varians/rosea*, *Staphylococcus aureus*, *Staphylococcus auricularis*, *Staphylococcus haemolyticus*, *Staphylococcus lentus*, *Morganella morganii*, *Pasteurella multocida*, *Pasteurella pneumotropica/haemolytica*, *Proteus* spp., *Pseudomonas putida*, *Salmonella enterica* ssp. *arizonae*, *Stenotrophomonas maltophilia* and *Vibrio parahaemolyticus* was evidenced. The presence in 8 animals of *Mycoplasma* spp. and in 1 animal with severe conjunctivitis of *Chlamydia* spp. was detected by PCR. *Candida* spp. was also isolated from two healthy animals.

**Conclusions:**

A clear predominance of Gram positive isolates in tortoises and Gram negative isolates in turtles was found. However, we cannot ascribe the observed difference to the diversity of animal species, as other factors, including especially different characteristics of the living environments, may play a role. Almost all bacterial species isolated may have clinical significance, mostly as opportunistic pathogens, both for humans and animals. That chelonians are often carrier of bacteria with zoonotic potential is a well-known fact, in particular with regard to *Salmonella* spp. Therefore, it is not surprising the detection of a strain of *Salmonella enterica* ssp. *arizonae* in the eye of one of the animals tested. Worthy of note is the finding of *Chlamydia* spp. in a severe case of conjunctivitis, though we cannot epidemiologically assess a cause-effect relationship between presence of chlamydia and disease.

**Electronic supplementary material:**

The online version of this article (doi:10.1186/s12917-015-0405-x) contains supplementary material, which is available to authorized users.

## Background

Chelonians are kept in captivity as pets or for commercial purpose. However, the progressive increase in the number of these animals which are in close contact with humans has not been matched with an appropriate increase in medical knowledge. Chelonians may have different common names, depending on their habitat. Turtles, as those who belong to the genera *Trachemys*, *Emydura* and *Pseudemys*, spends most of their life in the water, while tortoises, which includes the genus *Testudo*, are land-dwellers. Finally, *Pelomedusa subrufa* can be considered a terrapin and spends its time both on land and in water. Chelonians belong to the class of *reptilian*, order *testudines*. The suborder *Cryptodira* includes tortoises as *Testudo* spp., which belong to the family of *Testudinidae*, as well as turtles as *Trachemys scripta* and *Pseudemys* spp., which belong to the family of *Emydidae. Pelomedusa subrufa* and *Emydura subglobosa* belong to the *Pleurodira* suborder, *Pelomedusidae* and *Chelidae* families, respectively.

Chelonians are ectothermic animals and they need direct ultraviolet B (UV-B) irradiation to maintain optimal body temperature and vitamin D3 activation. The bactericidal property of UV radiation contribute to the control of cutaneous bacterial flora. Furthermore, inadeguated UV irradiation often results in a reduction of immune system activity [1]. In chelonians immune depression and vitamin deficiencies (hypovitaminosis A and D) are the main causes of metabolic disease that often occur with conjunctivitis [2]. Conjunctivitis is also a common symptom of upper respiratory tract disease (URTD), a widespread disease among chelonians, characterized by mild to severe rhinitis, nasal and ocular discharge, conjunctivitis, and periocular edema [3].

Knowledge of the conjunctival microbical flora, particularly those of pathological cases, is essential for treatment of eye diseases. The aim of this study was to investigate the bacterial and fungal flora present in the eyes of healthy and pathological chelonians and to compare findings in turtles with those in tortoises.

## Methods

### Animals

Thirty-four animals, belonging to the *Cryptodira* suborder (8 *Testudo graeca*, 8 *Testudo hermanni*, 1 *Testudo hosfieldi*, 1 *Testudo marginata*, 1 *Pseudemys* spp., and 13 *Trachemys scripta*) and to the *Pleurodira* suborder (1 *Pelomedusa subrufa* and 1 *Emydura subglobosa*), brought to the veterinary hospital of the Department of Veterinary-Medical Sciences of the University of Parma, underwent routine ophthalmic examination as part of a general health check and were subjected to microbiological examination. All the animals were captive animals and came from private collections of northern Italy, with the exception of an healthy wild *Trachemys scripta elegans* (n. 27) caught in the river Po. As reported in Table 1, four of the 34 animals showed bilateral conjunctivitis, but they were not affected by URTD or any other symptom. Moreover, another animal (n. 18) was weakened, with no specific symptoms. In all the diseased animals hypovitaminosis was excluded. The research comply with the current law of the European Union and Italy regarding the protection of animals used for experimental and other scientific purposes (Directive 86/609/EEC - D. L.vo 116/92) and was approved by the Ethics Committee on animal experimentation of the University of Parma.

**Table 1:** **Results of Nested-PCRs and cultures on eye swabs from healthy and diseased tortoises and turtles**

**Sample**	**Species**	**Tortoise**	**Turtle**	**Clinical signs**	***Chlamydia*** **PCR**	***Mycoplasma*** **PCR**	Gram positive isolates*								**Gram negative isolates** ^**§**^														***Candida*** **spp.**
							***A***	***B***	***C***	***D***	***E***	***F***	***G***	***H***	***I***	***J***	***K***	***L***	***M***	***N***	***O***	***P***	***Q***	***R***	***S***	***T***	***U***	***V***	
1	*T. graeca*	X		None			+		+																				
10	*T. graeca*	X		Conjunctivitis	+		+							+															
11	*T. hosfieldi*	X		Conjunctivitis		+								+															
13	*T. graeca*	X		None						+													+						
14	*T. hermanni*	X		Conjunctivitis			+							+															
15	*T. graeca*	X		None		+	+																						+
16	*T. graeca*	X		None			+							+															+
17	*T. graeca*	X		None			+							+															
18	*T. graeca*	X		Weakeness		+	+	+						+															
19	*T. hermanni*	X		None			+						+				+												
20	*T. hermanni*	X		None			+						+				+										+		
21	*T. hermanni*	X		None			+						+																
22	*T. hermanni*	X		None			+						+												+	+			
24	*T. graeca*	X		None									+								+								
25	*T. marginata*	X		None			+							+															
26	*T. hermanni*	X		None										+					+										
33	*T. hermanni*	X		None			+							+															
34	*T. hermanni*	X		None										+															
2	*T. scripta*		X	None		+																				+			
3	*T. scripta*		X	None																						+			
4	*T. scripta*		X	None		+																				+			
5	*P. subrufa*		X	None		+									+														
6	*E. subglobosa*		X	None		+									+	+													
7	*T. scripta*		X	None															+										
8	*T. scripta*		X	None															+							+			
9	*T. scripta*		X	None																+								+	
12	*T. scripta*		X	None											+														
23	*T. scripta*		X	Conjunctivitis							+							+											
27	*T. scripta*		X	None								+										+							
28	*T. scripta*		X	None																						+			
29	*T. scripta*		X	None																				+					
30	*T. scripta*		X	None		+									+	+													
31	*T. scripta*		X	None											+														
32	*Pseudemys* spp.		X	None																			+						

*Gram positive isolates: *A=Bacillus* spp.*; B=Kocuria varians/rosea; C=Staphylococcus aureus; D=Staphylococcus auricularis; E=Staphylococcus haemolyticus; F=Staphylococcus lentus; G=Staphylococcus sciuri; H=Staphylococcus xylosus.*

^§^Gram negative isolates: *I=Aeromonas hydrophila/caviae; J=Citrobacter freundii; K=Enterobacter cloacae; L=Morganella morganii; M=Ochrobactrum anthropic; N=Pasteurella multocida; O=Pasteurella pneumotropica/ haemolytica; P=Proteus* spp.*; Q=Pseudomonas luteola; R=Pseudomonas putida; S=Salmonella enterica* ssp. *arizonae; T=Sphingomonas paucimobilis; U=Stenotrophomonas maltophilia; V=Vibrio parahaemolyticus.*

### Culture

Samples were taken from the conjunctival sac of each eye with sterile swabs and bacterial and fungal investigation were immediately performed. Tryptose agar (Beckton Dickinson, Sparks, Maryland, USA) containing 5% of bovine erythrocytes, MacConkey agar (Beckton Dickinson), Sabouraud agar (Beckton Dickinson), Brain Heart Infusion broth (BHI) (Beckton Dickinson) and Mycoplasma agar (Beckton Dickinson) were inoculated and incubated in aerobic and/or microaerophilic (air with 5% CO_2_) atmosphere. Plates were inoculated and incubated for 24 hours at 37°C or for 48 hours at 35°C for bacteriological and micological investigation, respectively. The incubation of Mycoplasma agar plates in 5% CO_2_ air was extended to 7 days and plates were examined daily with a plate microscope. BHI broth is an enrichment medium and was inoculated to be subcultured in case of total absence of growth on agar plates. Bacterial isolates were identified using standard microbiological procedures, as growth and colonial characteristics, Gram staining, cellular morphology, catalase and oxidase reactions, coagulase test (Slidex Staph Plus, bioMérieux, Marcy-l’Etoile, France), haemolysin production. Species identification was carried out using the API biochemical test systems (bioMérieux, Marcy-l’Etoile, France), as well as conventional biochemical tests [4].

### DNA extraction

To detect the presence of *Mycoplasma* spp. or *Chlamydia* spp., one swab from each eye was placed in a 2 ml tube containing 400 μl of PBS and 1 mM EDTA, thoroughly vortexed and stored at −80°C until DNA extraction. For DNA extraction, each sample was thawed and thoroughly vortexed, swab was removed and the solution was transferred to a 1.5 ml tube containing Phase Lock Gel Light (Eppendorf, Hamburg, Germany). Four-hundred μl of phenol/chlorophorm (Invitrogen) were added and mixed by repeated inversion. After 10 min of incubation at RT, the tube was centrifuged (Microfuge 18 Centrifuge, Beckman Coulter) at 14000 rpm for 10 min at room temperature (RT). The acqueous phase was removed and transferred to a new eppendorf tube. Four-hundred μl of isopropanol and 40 μl of 3 M, pH 4.8, potassium acetate were added and mixed by repeated inversion. After 10 min of incubation at RT, the tube was centrifuged at 14000 rpm for 10 min, the surnatant was removed and the pellet gently washed with 500 μl of 70% ethanol. After centrifugation at 14000 rpm for 5 min the ethanol was completely removed and the pellet was resuspended in 50 μl of sterile deionized water. A 5 μl aliquot of this DNA suspension was used for PCR amplification.

### *Mycoplasma* genus-specific PCR

**Figure 1:**
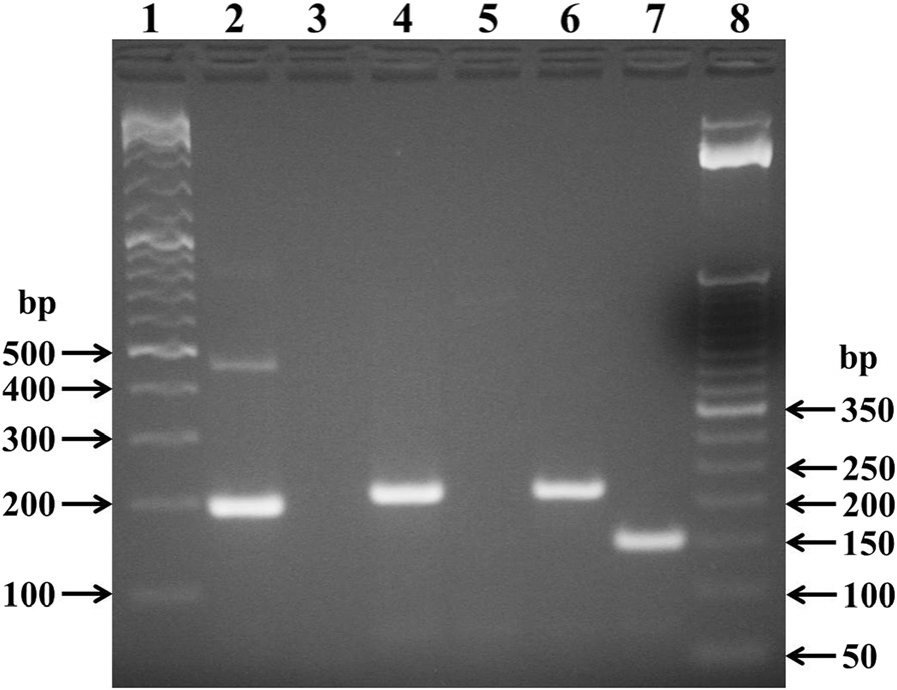
**Representative electrophoresis of PCR products for the detection of** ***Mycoplasma*** **spp. in eye swabs samples.** Lane 1: DNA size markers (GeneRuler DNA Ladder Mix, Fermentas); lane 2: positive control (*Mycoplasma bovis* DNA); lane 3: negative control (no template); lanes 4, 6 and 7: positive samples (animals n. 2, 4 and 5, respectively); lane 5: negative sample (animal n. 3); lane 8: DNA size markers (TrackIt 50 bp DNA Ladder, Life Technologies).

A nested PCR that target a conserved intergenic spacer region between the 16S and 23S rRNA gene of *Mycoplasma* spp. was used [5]. External primers for primary PCR were F1-sense (5′-ACACCATGGGAGCTGGTAAT-3′) and R1-antisense (5′-CTTCATCGACTTTCAGACCCAAGGCAT-3′), whilst internal primers for secondary PCR were F2-sense (5′-GTTCTTTGAAAACTGAAT-3′) and R2-antisense (5′-GCATCCACCAAAAACTCT-3′). Depending on the different mycoplasma species, the expected amplicons lengths ranged from 369 to 681 bp for the first PCR and from 145 to 237 bp for the second PCR. Five microliter of the extracted DNA were added to a reaction mixture (total volume of 50 μl) containing 2.5 mM MgCl_2_, PCR buffer (20 mM Tris–HCl [pH 8.4], 50 mM KCl), 0.2 mM deoxynucleoside triphosphates (Invitrogen) 0.5 μM of external primers, and 1.5 U of *Taq* DNA polymerase (Invitrogen). Amplification was performed using a PTC 150 thermal cycler (MJ research, Waltham, MA, USA) with the following settings: one cycle of 10 min at 94°C, followed by 30 cycles of denaturation at 94°C for 30 sec, annealing at 55°C for 2 min, extension at 72°C for 2 min, and by one cycle of final extension at 72°C for 5 min. The product (2 μl) was used in secondary PCR, performed in the same manner as the primary PCR, but with internal primers instead of external ones. The amplified products were electrophoresed on a 2.5% agarose gel and visualized by ethidium bromide fluorescence. From positive specimens a fragment of around 200 bp, or alternatively of around 150 bp, was amplified after the second round PCR (Figure 1). *Mycoplasma bovis* DNA was used as positive control. Deionized water was used as negative control (no template control). Positive and negative controls were included for each round of amplification. Samples analysis was repeated twice.

### *Chlamydia* genus-specific PCR

**Figure 2:**
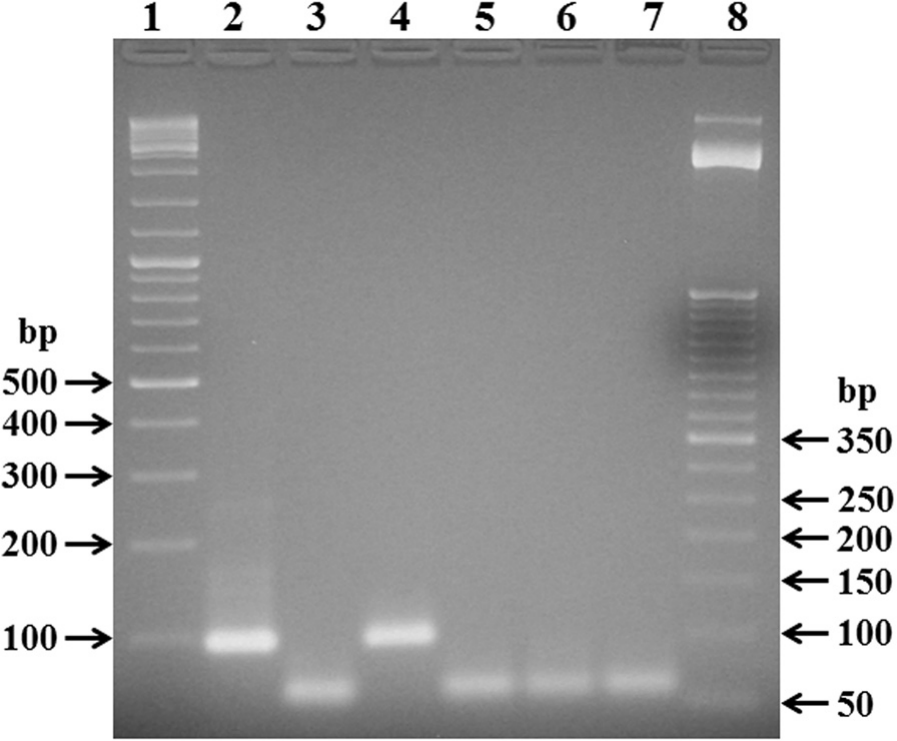
**Representative electrophoresis of PCR products for the detection of** ***Chlamydia*** **spp. in eye swabs samples.** Lane 1: DNA size markers (GeneRuler DNA Ladder Mix, Fermentas); lane 2: positive control (*Chlamydia psittaci* DNA); lane 3: negative control (no template); lane 4: positive sample (animal n. 10); lanes 5, 6 and 7: negative samples (animals n. 11, 12 and 13, respectively); lane 8: DNA size markers (TrackIt 50 bp DNA Ladder, Life Technologies). In the negative control and samples the lower band represent dimers of primers.

A nested PCR that amplifies a portion of the MOMP gene of *Chlamydia* spp. was used. External primers for primary PCR were CHL2A (5′-GAAAAAACTCTTRAARTCGG-3′) and CHL2B (5′-CGNANGCTWATRGCRTCRCACCAAG-3′), whilst internal primers for secondary PCR were CHL2C (5′-TGCCTGTRGGGAAYCCWKCTGAWCCAAG-3′) and CHL2D (5′-CAAGTNCNRCAAGGATCRCAAGGATC-3′) [6]. Five microliter of the extracted DNA were added to a reaction mixture (total volume of 50 μl) containing 2.5 mM MgCl_2_, PCR buffer (20 mM Tris–HCl [pH 8.4], 50 mM KCl), 0.2 mM deoxynucleoside triphosphates (Invitrogen) 0.5 μM of external primers, and 1.5 U of *Taq* DNA polymerase (Invitrogen). Amplification was performed using a PTC 150 thermal cycler (MJ research, Waltham, MA, USA) with the following settings: one cycle of 3 min at 94°C, followed by 35 cycles of denaturation at 94°C for 20 sec, annealing at 45°C for 20 sec, extension at 72°C for 30 sec. The product (2 μl) was used in secondary PCR, performed in the same manner as the primary PCR, but with internal primers instead of external ones. The amplified products were electrophoresed on a 2.5% agarose gel and visualized by ethidium bromide fluorescence. Specimens were regarded as positive if an appropriately sized band (90 bp) was present on second round PCR (Figure 2). *Chlamydia psittaci* DNA was used as positive control. Deionized water was used as negative control (no template control). Positive and negative controls were included for each round of amplification.

### DNA sequencing

Amplicons were purified and extracted from a 2% agarose gel by a spin column technique (JETQUICK Gel Extraction Spin Kit, Genomed, Löhne, Germany). Sequences were obtained by cycle sequencing on an ABI 3730XL sequencing machine using F2 and R2 primers. GeneBank sequence alignment function (BLAST) was used for sequence analysis.

### Statistical analysis

Statistical analysis was performed using the Fisher’s exact test.

## Results

All the cultured samples showed bacterial growth on agarized media. Therefore, BHI broth cultures were not used for subculturing. Among fungi, only *Candida* spp. was detected in 2 healthy tortoises, both *T. graeca* (Table 1). Thirteen animals (38%), almost all turtles (10/13), harboured a single bacterial species as sole isolate. Seventeen animals (50%) harboured 2 different species of bacteria, 2 animals (6%) harboured 3 species and 2 animals (6%) harboured 4 species (Table 1). The detection of multiple bacterial infection was clearly greater (p = 0.011) in tortoises (17 out of 18 animals) compared to turtles (9 out of 16 animals). Besides *Bacillus* spp. (13 isolates), the most frequently isolated bacterial species were *Staphylococcus xylosus* (10 isolates), *Sphingomonas paucimobilis* (6 isolates), *Staphylococcus sciuri* and *Aeromonas hydrophila/caviae* (each 5 isolates), *Ochrobactrum anthropi* (3 isolates), and finally *Citrobacter freundii*, *Enterobacter cloacae* and *Pseudomonas luteola* (each 2 isolates). Only one isolate of *Kocuria varians/rosea*, *Staphylococcus aureus*, *Staphylococcus auricularis*, *Staphylococcus haemolyticus*, *Staphylococcus lentus*, *Morganella morganii*, *Pasteurella multocida*, *Pasteurella pneumotropica/haemolytica*, *Proteus* spp., *Pseudomonas putida*, *Salmonella enterica* ssp. *arizonae*, *Stenotrophomonas maltophilia* and *Vibrio parahaemolyticus* was evidenced (Table 1). Mycoplasma cultures were negative for all the animals. By PCR we detected eight animals, of which 1 diseased and 2 healthy tortoises and 5 healthy turtles, positive for *Mycoplasma* spp., whereas only one tortoise with conjunctivitis was positive for *Chlamydia* spp. (Table 1). Sequencing analysis, performed on *Mycoplasma* PCR products, confirmed that all the amplified sequences belong to the *Mycoplasma* genus (sequences are reported in an additional file [see Additional file 1]; BLAST analysis results are not shown).

## Discussion

The number of people who raise reptiles as pets has increased, but information about microbiological is limited. Among reptiles, pet turtles and tortoises are also becoming increasingly popular. The animals involved in this survey are almost all captive chelonians, that are routinely or occasionally subjected to medical examination.

Except the presence of *Bacillus* spp. (13 isolates), *Staphylococcus xylosus* was the most frequent isolate in tortoises (10 isolates). *Sphingomonas paucimobilis* and *Aeromonas hydrophila/caviae* were likewise the most frequent isolates in turtles (6 and 5 isolates, respectively).

From Table 1 a clear predominance of Gram positive isolates in tortoises and Gram negative isolates in turtles can be noticed (p < 0.001). A preponderance of Gram positive species was already observed by the Authors in the normal conjunctival bacterial flora of green iguana, in accordance with data reported for other terrestrial animal species [7]. The prevalence of Gram negative isolates in water turtles is also a not surprising result. Captive water turtles, as those included in the present study, often live in small water volumes of aquaria and ponds, where fecal matter and other waste material tend to be concentrated. Therefore, these animals live immersed in a medium were proliferation of enteric bacteria is favoured. To date, little is known about the microbial communities or potential pathogens associated with aquarium water. However, in a survey of Smith et al. [8] the bacterial community of ornamental fish aquarium freshwater was characterized by molecular methods. In accordance with our results, they found in their samples the presence of *Aeromonas* and *Vibrio* genera, which include species that can become pathogenic for fish as for turtles in stressful conditions. On the contrary, they did not found the presence of *Salmonella* spp. This is not unexpected, as it is well known that reptiles are frequently carriers of *Salmonella* spp. [9]. However, it was reported that also bacteria belonging to *Salmonella* genus, as well as *Aeromonas* and *Vibrio* genera, can be present in home aquaria and probably be responsible of illness in humans due to exposure to ornamental fish [10].

The occurrence of conjunctivitis was higher in tortoises than in turtles: three out of eighteen tortoises (n. 10, 11 and 14) and one out of sixteen turtles (n. 23), showed bilateral conjunctivitis (Table 1). However, the data is not statistically significant (p = 0.282) and to be confirmed would require a greater number of samples. As already it is clear looking at the data, the statistical analysis confirms the absence of association (p = 0.398) between the presence of conjunctivitis and the presence of infection with multiple bacterial species. Bacterial flora of tortoises 11 and 14 did not differed from that of other healthy tortoises. In these animals, as well as in tortoise 10, the only common isolate was represented by *Staphylococcus xylosus*. However, tortoise 10 was the only one in which it was possible to detect the presence of *Chlamydia* spp. by PCR. Unlike in the other clinical cases, in this animal conjunctivitis was particularly severe and the animal was not able to open the eyelids themselves. This finding may indicate a role of chlamydia in inducing severe ocular disease. As reviewed by Corsaro and Venditti [11], *Chlamydia* or *Chlamydia*-like organisms infections have occasionally been reported in reptiles, including both turtles and tortoises, and almost all the reported cases concerned captive animals. Common symptoms are lethargy, anorexia and chronic respiratory disease. However, almost all *Chlamydiaceae* species are able to infect the conjunctiva of their vertebrate host and, with regard to reptiles, exudative conjunctivitis attributed to *Chlamydia* spp. infection was reported in farmed juvenile crocodiles [11,12].

On the other hand, it is noteworthy that bacterial isolates from turtle 23, which is the only acquatic turtle showing conjunctivitis, that is *Staphylococcus haemolyticus* and *Morganella morganii*, were not found in any of the other turtles. *Staphylococcus haemolyticus* belong to the group of coagulase-negative staphylococci (CNS). CNS are a major component of the normal microflora of the human skin and are mostly considered to be saprophytes. However, *S. haemolyticus* can be also an opportunistic pathogen and is the second most frequently encountered species of CNS in human clinical infections [13]. *S. haemolyticus* is a frequently isolated pathogen in dairy cows and small ruminants and can be sporadically involved in subclinical mastitis [14]. Less is known about the relation of this bacterium and other animal species. Instead, it is well known the ability of *S. haemolyticus* to acquire multi-antibiotic resistance [15]. In our case, *S. haemolyticus*, as well as *S. lentus*, was found in acquatic animals, while, on the contrary, all the other species of staphylococci were detected in animals with terrestrial habitat. One isolate of *S. aureus* was found in tortoise n. 1. *S. aureus* may be involved in case of ocular disease induced by hypovitaminosis in tortoises [16], but in our case the animal was health. *M. morganii* is a Gram negative facultative anaerobe, belonging to the *Enterobacteriaceae* family. It is commonly found in the environment and as normal flora in the intestinal tracts of humans, mammals other than humans and reptiles and is considered an opportunistic bacterium. In human, despite its wide distribution, is an uncommon cause of nosocomial infections in adults and is mostly isolated from urinary tract or wound infections, as well as can be involved in neonatal sepsis [17]. A case of postoperative bacterial endophtalmitis caused by *M. morganii* was also reported [18]. In animals, *M. morganii* was isolated from mixed infections of the marine mammal *Dugong dugon* [19] and was reported as one of the most common pathogens identified in human secondary wound infections following snakebites [20]. Recently, a sporadic case of fatal infection in chickens, caused by a highly pathogenic *M. morganii* strain, was also reported [21]. Our isolate of *Morganella morganii* was resistant to a wide variety of antibiotics suitable for chelonians treatment (data not showed). The presence of multiple drug resistance is significant, since the bacterium can, although rarely, cause the occurrence of zoonotic disease, particularly in weak persons.

The presence of *Mycoplasma* negative cultures, also from those animals that resulted positive to *Mycoplasma* PCR, is not a surprising result [22] as mycoplasmas have many different and often fastidious requirements for growth. The seven days incubation time may have further reduced the probability of isolating slow growing mycoplasmas. In *Mycoplasma* PCR we found an occasional unspecific amplification, outside the 145–237 bp range of positivity, in the negative control. This could be due to an external contamination of the water used in place of template in the negative control. Conversely, we exclude the contamination of the reaction mix, because such unspecific amplification has never occurred to any of the samples tested. However, to doubtless confirm that the amplified sequences belong to the *Mycoplasma* genus, we repeated twice the *Mycoplasma* PCR and we performed sequencing analysis on *Mycoplasma* PCR products. We found a higher percentage of *Mycoplasma* PCR positive samples in turtles compared to tortoises (Table 1). Mycoplasmas are frequently found in reptile species, particularly in case of chelonians, and some of them are part of the commensal bacterial flora of the host. However, *Mycoplasma agassizii* and *Mycoplasma testudineum* can be involved in the URTD in tortoises [23]. In particular, the most frequently reported agent of URTD in tortoises is *Mycoplasma agassizii* [24,25]. We found by PCR the presence of *Mycoplasma* spp. in 23,5% of the animals (16.7% for tortoises and 31,3% for turtles). Our result for the testudo genus is similar to those obtained by Soares et al. [25], who reported a prevalence of 15.8% for *Mycoplasma agassizii* in captive tortoises (*Testudo* spp.) in the United Kingdom. In Italy, in particular on the island of Sardinia, a higher prevalence of *Mycoplasma* spp. (37%) was found by Lecis et al. [24] in tortoises belonging to the *Testudo* genus. However, in addition to the different geographical origin of the animals, Lecis et al. [24] used wild animals, while in our case, with only one exception (animal n. 27), the animals came from private collections. Moreover, they have analyzed oral and cloacal swabs and used a different technique of DNA extraction and amplification. Considering our study, two results emerge: the fact that the detection of *Mycoplasma* spp. is almost double in turtles than tortoises, but with a number of observations that does not allow to assign statistical significance to the data (p = 0.196), and that the presence of *Mycoplasma* spp. was not statistically associated with the presence of conjunctivitis (p = 0.424). It would be interesting to assess whether, in case of URTD with simultaneous presence of conjunctivitis, such an association could occur.

Regarding the presence of *Candida* spp., which we have found in 2 healthy tortoises, the occurrence of yeasts in reptiles, especially in those who predominantly consuming vegetable diets, has been reported, and different *Candida* species have been isolated from tortoises belongin to the *Testudinidae* family [26]. Usually reptiles colonized with yeasts do not reveal any symptoms, as in our case, and we consider this finding not worthy of further investigation for the present study. However, representatives of the *Testudinidae* are frequently kept as pets, as indeed in the case of our tested animals, and could play a role in transmission of yeasts to human beings, especially in immunocompromised hosts, children and elderly or ill persons who are predisposed to candidosis.

*Stenotrophomonas maltophilia* causes many opportunistic infections as sepsis, pneumonia, urinary tract infection, meningitis, endocarditis, septic arthritis, and peritonitis. It has also been noted to be a pathogen in many ocular infections, including conjunctivitis, keratitis, dacryocystitis, cellulitis, infected scleral buckles, and endophthalmitis, as reviewed by Chang et al. [27]. However, in our case the bacterium was isolated from an healthy animal.

## Conclusions

A clear predominance of Gram positive isolates in tortoises and Gram negative isolates in turtles was found. However, we cannot ascribe the observed difference to the diversity of animal species, as other factors, including especially the different characteristics of the living environments, may play a role. Almost all bacterial species isolated may have clinical significance, mostly as opportunistic pathogens, both for humans and animals. That chelonians are often carrier of bacteria with zoonotic potential is a well-known fact, in particular with regard to *Salmonella* spp. Therefore, it is not surprising the detection of a strain of *Salmonella enterica* ssp. *arizonae* in the eye of one of the animals tested. Worthy of note is the finding of chlamydia in a severe case of conjunctivitis, though we cannot epidemiologically assess a cause-effect relationship between the presence of chlamydia and disease.

### Availability of supporting data

The data set supporting the results of this article is included within the article (and its additional file).

## Additional file

Additional file 1:
***Mycoplasma*** **PCR products sequences.** In the Additional file 1 the plain format sequences of the amplicons obtained from *Mycoplasma* PCR positive samples and control are reported.

## References

[1] Rossi JV, Mader DR (2006). Biology and husbandry. Reptile medicine and surgery.

[2] Williams DL (2012). Ophthalmology of exotic pets.

[3] Origgi FC, Jacobson ER (2000). Diseases of the respiratory tract of chelonians. Vet Clin North Am Exot Anim Pract.

[4] Quinn PJ, Carter ME, Markey BK, Carter GR (1994). Bacterial pathogens: microscopy, culture and identification. Clinical veterinary microbiology.

[5] Harasawa R, Uemori T, Asada K, Kato I, Kahane I, Adoni A (1993). Sensitive detection of mycoplasmas in cell cultures by using two-step polymerase chain reaction. Rapid diagnosis of mycoplasmas.

[6] Ong GM, Coyle PV, Barros D’Sa AAB, McCluggage WG, Duprex WP, O’Neill HJ (2001). Non-detection of *Chlamydia* species in carotid atheroma using generic primers by nested PCR in a population with a high prevalence of *Chlamydia pneumoniae* antibody. BMC Infect Dis.

[7] Taddei S, Dodi PL, Di Ianni F, Cabassi CS, Cavirani S (2010). Conjunctival flora of clinically normal captive green iguanas (*Iguana iguana*). Vet Rec.

[8] Smith KF, Schmidt V, Rosen GE, Amaral-Zettler L (2012). Microbial diversity and potential pathogens in ornamental fish aquarium water. PLoS One.

[9] Warwick C, Lambiris AJ, Westwood D, Steedman C (2001). Reptile-related salmonellosis. J R Soc Med.

[10] Weir M, Rajić A, Dutil L, Cernicchiaro N, Uhland FC, Mercier B (2012). Zoonotic bacteria, antimicrobial use and antimicrobial resistance in ornamental fish: a systematic review of the existing research and survey of aquaculture-allied professionals. Epidemiol Infect.

[11] Corsaro D, Venditti D (2004). Emerging chlamydial infections. Crit Rev Microbiol.

[12] Huchzermeyer FW, Langelet E, Putterill JF (2008). An outbreak of chlamydiosis in farmed Indopacific crocodiles (*Crocodylus porosus*). J S Afr Vet Assoc.

[13] Balows A, Hausler WJ, Herrmann KL, Isenberg HD, Shadomy HJ, Balows A (1991). Staphylococcus. Manual of clinical microbiology.

[14] Leitner G, Sela S, Hammer-Muntz O, Zivotofsky D, Weisblit L, Chaffer M (2009). Outbreak of subclinical mastitis in a flock of dairy goats associated with atypical *Staphylococcus haemolyticus*. J Dairy Res.

[15] de Allori MC, Jure MA, Romero C, de Castillo ME (2006). Antimicrobial resistance and production of biofilms in clinical isolates of coagulase-negative *Staphylococcus strains*. Biol Pharm Bull.

[16] Holt PE, Cooper JE, Needham JR (1979). Diseases of tortoises: a review of seventy cases. J Small Anim Pract.

[17] Chang HY, Wang SM, Chiu NC, Chung HY, Wang HK (2011). Neonatal *Morganella morganii* sepsis: a case report and review of the literature. Pediatr Int.

[18] Zaninetti M, Baglivo E, Safran AB (2003). *Morganella morganii* endophthalmitis after vitrectomy: case report and review of the literature. Klin Monbl Augenheilkd.

[19] Nielsen KA, Owen HC, Mills PC, Flint M, Gibson JS (2013). Bacteria isolated from dugongs (*Dugong dugon*) submitted for postmortem examination in Queensland, Australia, 2000–2011. J Zoo Wildl Med.

[20] Chen CM, Wu KG, Chen CJ, Wang CM (2011). Bacterial infection in association with snakebite: a 10-year experience in a Northern Taiwan medical center. J Microbiol Immunol Infect.

[21] Zhao C, Tang N, Wu Y, Zhang Y, Wu Z, Li W (2012). First reported fatal *Morganella morganii* infections in chickens. Vet Microbiol.

[22] Pinard CL, Brightman AH, Yeary TJ, Everson TD, Cox LK, Chengappa MM (2002). Normal conjunctival flora in the North American opossum (*Didelphis virginiana*) and raccoon (*Procyon lotor*). J Wildl Dis.

[23] Farkas SL, Gál J (2009). Adenovirus and mycoplasma infection in an ornate box turtle (*Terrapene ornata ornata*) in Hungary. Vet Microbiol.

[24] Lecis R, Paglietti B, Rubino S, Are BM, Muzzeddu M, Berlinguer F (2011). Detection and characterization of *Mycoplasma* spp. and *Salmonella* spp. in free-living European tortoises (*Testudo hermanni, Testudo graeca, and Testudo marginata*). J Wildl Dis.

[25] Soares JF, Chalker VJ, Erles K, Holtby S, Waters M, McArthur S (2004). Prevalence of *Mycoplasma agassizii* and Chelonian herpesvirus in captive tortoises (*Testudo* sp.) in the United Kingdom. J Zoo Wildl Med.

[26] Milde K, Kostka V, Kaleta EF, Willems H, Jäger C (2000). Multiplex-PCR-based differentiation and characterization of *Candida*-isolates derived from tortoises (*Testudinidae*). Vet Microbiol.

[27] Chang JS, Flynn HW, Miller D, Smiddy WE (2013). *Stenotrophomonas maltophilia* endophthalmitis following cataract surgery: clinical and microbiological results. Clin Ophthalmol.

