# Supplementary material for: Conjunctival flora of clinically normal and diseased turtles and tortoises
Source: BMC Vet Res. 2015 Apr 10;11:91. doi: 10.1186/s12917-015-0405-x (PMC4397695; doi:10.1186/s12917-015-0405-x)
Supplement: Additional file 1: — Mycoplasma PCR products sequences. In the Additional file 1 the plain format sequences of the amplicons obtained from Mycoplasma PCR positive samples and control are reported. [file 12917_2015_405_MOESM1_ESM.pdf]

Sequences of the amplicons obtained from *Mycoplasma* PCR positive samples and control. The reported sequences were used for BLAST analysis.

**Sample**	**Sequence**	**Primer**	**Left Clip**	**Right Clip**	**Length**
Turtle n. 2	ATCAAATATATTTTGTGAAATAATTTTGATCCGAGTTGTGTCTATACATGGGTATAGACACTATAAATACCTTAAGAATACATCAAAACAAAAAAACATTTTTTTTAAATAGGAAAAAATAGTTACTTATACTTTTAAATAAGCAAGAGTTTTTGGTGGATGCAGAGTTTTTGGGGGATGCAGAGATT	F2-sense	16	203	187
Turtle n. 4	ACATCAAAATATATTTTGTGAAATAATTTTGATCCGAGTTGTGTCTATACATGGGTATAGACACTATAAATACCTTAAGAATACATCAAAACAAAAAAACATTTTTTTTAAATAGGAAAAAATAGTTACTTATACTTTTAAATAAGCAAGAGTTTTTGGTGGATGCAGAGTTTTTGGTGGATGCAGAGTTTTTGGTGGATGCAGAGTTT	F2-sense	14	222	208
Turtle n. 5	CAAGTTTAGATCAACCTATAGAATATATCATACAATAGACAAACAATAGGTCTTATACTACTATTAAACAAGATAAGAGTTTTTGGTGGATGCA	F2-sense	26	119	93
Turtle n. 6	TTATTAATATTTCAAGTTTAGATCAACCTATAGAATATATCATACAATAGACAAACAATAGGTCTTATACTACTATTAAACAAGATAAGAGTTTTTGGTGGATGCCGTAA	F2-sense	10	119	109

Tortoise n. 11	TACGACATCAAATATATTTTGTGAAATAATTTTGATCCGAGTTAAGACCAATTATTGGTCTTTATAAATACCTTAAGAATACATCAAAAAACATTTTTTTTAATAGGAAAAAATAGTTACTTAAACTTTTAAATAAGCAAGAGTTTTTGGTGGATGATGA	F2-sense	10	169	159
Tortoise n. 15	ACGACATCAAATATATTTTGTGAAATAATTTTGATCCGAGTTAAGACCAATTATTGGTCTTTATAAATACCTTAAGAATACATCAAAAAACATTTTTTTTAATAGGAAAAAATAGTTACTTAAACTTTTAAATAAGCAAGAGTTTTTGGTGGATGCTGGC	F2-sense	10	169	159
Tortoise n. 18	TACGACATCAAATATATTTTGTGAAATAATTTTGATCCGAGTTAAGACCAATTATTGGTCTTTATAAATACCTTAAGAATACATCAAAAAACATTTTTTTTAATAGGAAAAAATAGTTACTTAAACTTTTAAATAAGCAAGAGTTTTTGGTGGATGATGA	F2-sense	8	167	159
Turtle n. 30	ATACGACATCAAATATATTTTGTGAAATAATTTTGATCCGAGTTGTGTCTATACATGGGTATAGACACTATAAATACCTTAAGAATACATCAAAACAAAAAAACATTTTTTTTAAATAGGAAAAAATAGTTACTTATACTTTTAAATAAGCAAGAGTTTTTGGTGGATGAAGA	F2-sense	8	180	172
M. bovis (Pos. Ctrl.)	CACGACATCAAAAATCAAATTAATGGTTAATTTGTTTTGATTCATCGAGTAAGTCATATTTAATATGATTCATTGAAATGTCTTAAAATACACATCTAAAACTAACAACAATAGGAAAATACTACTTTTAAATAAGGAAGAGTTTTTGGTGGATGCA	F2-sense	15	171	156
